# A Hydrogen-Based Bacterial–Algal Biofilm for Winter Nutrient Mitigation in a Paddy Soil Mesocosm: A Proof-of-Concept Study

**DOI:** 10.3390/microorganisms14102230

**Published:** 2026-10-03

**Authors:** Si Pang, Naxin Cui, Min Cai, Li Zhou, Yu Tian, Xu Zhang

**Affiliations:** 1 Eco-Environmental Protection Research Institute, Shanghai Academy of Agricultural Sciences, Shanghai 201403, China; pangsi@saas.sh.cn (S.P.);; 2 Shanghai Engineering Research Centre of Low-Carbon Agriculture, Shanghai 201403, China; 3 State Key Laboratory of Urban-Rural Water Resources and Environment, Harbin Institute of Technology, Harbin 150090, China

**Keywords:** ecological wetland, hydrogen, bacteria–algal biofilm, eutrophic water treatment, 16S full-length sequencing

## Abstract

The limited nitrogen and phosphorus concentration reduction performance of wetlands during cold weather is a global concern. To address the challenge of low nutrient removal efficiency in winter, this study introduces a hollow fiber membrane module integrated into paddy soil, enabling the in situ formation of a hydrogen-based bacterial–algal biofilm. Over a 60-day experiment at water temperatures of 6.2–15.0 °C, the biofilm-enhanced system (HS) reduced aqueous ${\mathrm{NH}}_{4}^{+}$-N from 25 mg/L to 0.2 mg/L by day 30 and maintained this low level through day 60, whereas the S system still had 5.5 mg/L ${\mathrm{NH}}_{4}^{+}$-N. Moreover, the HS system consistently maintained lower ${\mathrm{NO}}_{3}^{-}$-N and total phosphorus (TP) concentrations than the S system. Microbial communities were characterized using third-generation 16S full-length sequencing and 18S rRNA V4 amplicon sequencing. The biofilm was dominated by *Geminocystis_PCC_6308*, *Rikenellaceae_RC9_gut_group*, and *Rhodobacter*, while eukaryotic green algae, mainly *Botryococcus* sp., were also abundant. In the paddy soil, *Terrimonas* and *unclassified_AKYH767* were identified as potential key taxa associated with nitrogen and phosphorus concentration reduction. Overall, the hydrogen-based bacterial–algal biofilm effectively improves the nitrogen and phosphorus concentration reduction of paddy soil wetlands, offering a promising strategy for cold-weather eutrophic water remediation.

## 1. Introduction

The escalating eutrophication of surface waters, driven by agricultural non-point source pollution and inadequate wastewater treatment, has emerged as a pressing global environmental challenge [1,2]. Wetlands as nature-based solutions, have been widely recognized for their cost-effectiveness and ecological sustainability in mitigating nutrient loads from various sources [3]. The nutrient removal mechanisms in wetlands are governed by a complex interplay among substrate adsorption, plant uptake, and, most critically, microbial metabolic processes including nitrification, denitrification, and phosphorus accumulation [4]. However, due to the stringent environmental requirements of functional microorganisms, the nutrient removal efficiency of wetlands is subject to significant seasonal fluctuations, with particularly low denitrification efficiency during winter.

From a microbiological perspective, low temperatures impose multifaceted constraints on the microbial communities residing in the soil matrix and water column. The activity of nitrifiers, such as ammonia-oxidizing bacteria (AOB) and nitrite-oxidizing bacteria (NOB), is significantly inhibited below 15 °C, resulting in incomplete nitrification and ammonium accumulation [5]. Concurrently, the efficiency of denitrification, a key process for permanent nitrogen removal via gaseous N_2_ emissions, is restricted by the reduced enzymatic kinetics of nitrate reductases [6]. Moreover, the bioavailability of electron donors for denitrification, primarily derived from soil organic matter mineralization, declines sharply under cold stress due to reduced microbial metabolic rates and limited substrate diffusion [7]. These combined limitations often result in effluent water quality failing to meet regulatory standards, necessitating the development of innovative, low-temperature adaptation strategies that bypass the constraints of natural biological processes.

Symbiotic consortia of microalgae and bacteria possess the ability to adsorb and transform pollutants, representing a novel bioremediation approach. Such consortia are commonly found in biofilms attached to plants, sediments, and stone surfaces [8]. Microalgae assimilate inorganic nitrogen and phosphorus [9], whereas heterotrophic and autotrophic bacteria contribute to nitrogen removal via multiple biochemical pathways—including assimilation, anaerobic ammonium oxidation, nitrification, and denitrification [10]. This complementary interaction between microalgae and bacteria significantly enhances the concurrent removal of nitrogen and phosphorus from aqueous systems. In recent years, bacterial–algal biofilm technology has emerged as a promising approach for enhanced nitrogen and phosphorus removal in environmental applications. Zhang et al. [11] employed a hybrid bacterial–Chlorella symbiotic system in a membrane-aerated biofilm reactor (MABR) to treat aquaculture wastewater, achieving total nitrogen (TN) and total phosphorus (TP) removal rates of 58% and 65%, respectively. Another advantage of bacterial–algal biofilms is that, compared to MABRs with bacterial biofilms alone, TN removal efficiency is significantly improved regardless of the chemical oxygen demand (COD)/N ratio [12]. Despite these advantages, the efficient conversion of ammonium nitrogen (${\mathrm{NH}}_{4}^{+}$-N) to nitrate nitrogen (${\mathrm{NO}}_{3}^{-}$-N) by nitrification does not eliminate nitrogen from the system. Without a potent electron donor for subsequent denitrification, the final effluent often retains high TN due to the accumulation of nitrate, limiting the holistic nitrogen removal efficacy of these biofilms.

To overcome the limitation of nitrate accumulation, hydrogen gas (H_2_) has emerged as an ideal electron donor for autotrophic denitrification. As a clean and non-toxic reductant, H_2_ is oxidized by hydrogenotrophic denitrifiers to reduce ${\mathrm{NO}}_{3}^{-}$-N. Compared with traditional heterotrophic denitrification using external organic carbon sources (e.g., methanol or sodium acetate), H_2_ offers distinct advantages, including lower operational costs, minimal biomass production (reducing sludge disposal issues), and the elimination of secondary organic carbon pollution in treated effluents [13,14]. However, the low aqueous solubility of H_2_ renders conventional bubble aeration highly inefficient. To circumvent this, hollow fiber membranes (HFMs) were used as a gas-delivery vehicle, in which H_2_ diffuses without bubble formation directly from the membrane lumen to the biofilm attached on the outer surface, achieving nearly 100% gas utilization efficiency [15]. Building on this concept, subsequent studies have successfully applied hydrogen-based membrane biofilm reactors for treating nitrate-contaminated drinking water, groundwater, and secondary municipal effluents, consistently achieving over 90% removal of both nitrate and TN [13,16,17].

While H_2_-based membrane biofilms and algal–bacterial consortia have been studied separately, their integration into paddy soil for winter nutrient removal, and the associated soil–biofilm microbial interactions, remain poorly characterized. Paddy soil, as a unique agroecosystem component, harbors rich microbial diversity shaped by long-term plant–soil interactions [18]. However, scant attention has been paid to the combination of H_2_-based autotrophic denitrification and microalgal–bacterial symbiosis within wetland paddy soils, particularly under low-temperature conditions where both nitrification and denitrification are constrained. Furthermore, the response of the native soil microbiome to the establishment of this functional biofilm at the soil–water–membrane interface, as well as the microbial community shifts induced by H_2_ supply and algal colonization, has not been characterized via high-resolution sequencing.

To address these knowledge gaps, this study introduces a novel HFM module directly integrated into paddy soil, fostering the in situ formation of an H_2_-based bacterial–algal symbiotic biofilm. The primary objectives of this work are to: (1) evaluate the performance of this enhanced paddy-soil mesocosm for removing ${\mathrm{NH}}_{4}^{+}$-N, ${\mathrm{NO}}_{3}^{-}$-N, and TP compared to conventional paddy soil mesocosms under winter conditions (<15 °C); (2) elucidate the microbial composition and distribution of the indigenous biofilm and the underlying soil using third-generation 16S full-length sequencing; and (3) characterize the microbial community composition and identify potential key taxa associated with nutrient removal. This study is designed as a proof-of-concept investigation. The objective is to evaluate the feasibility of integrating a H_2_-based bacterial–algal biofilm into a paddy soil mesocosm for winter nutrient mitigation, rather than to provide a definitive assessment of long-term, full-scale performance.

## 2. Materials and Methods

### 2.1. Experimental Materials

Paddy soil was collected from the top 0~20 cm layer of a rice paddy field at the Zhuanghang Experimental Station of the Shanghai Academy of Agricultural Sciences (121°23′15″ E, 30°53′24″ N), following the harvest season. The soil was transported to the laboratory, homogenized, and passed through a 2 mm sieve to remove plant residues, stones, and macrofauna. The initial physicochemical properties of the soil were determined as follows: TP 1.07 g/kg, TC 2.73%, TN 2.53 g/kg, and pH 6.8 (measured in a 1:2.5 soil:water slurry).

Six clean white plastic square buckets (length 45 cm, width 32 cm, height 34 cm, effective volume approximately 35 L) were used as experimental containers. Each bucket was evenly covered with a 7 cm layer of the prepared paddy soil at the bottom, carefully leveled, and gently compacted. Subsequently, each bucket was filled with 23 cm depth of simulated eutrophic water, resulting in a total working volume of approximately 25 L per mesocosm. The simulated high-ammonia eutrophic water was prepared by dissolving 117.5 mg/L ammonium sulfate ((NH_4_)_2_SO_4_) and 12.5 mg/L potassium dihydrogen phosphate (KH_2_PO_4_) in tap water that had been dechlorinated by standing for 48 h. These concentrations were selected to mimic moderately to heavily eutrophic surface waters commonly found in agricultural drainage systems in the Yangtze River Delta region. After the addition of the simulated water, the system was allowed to equilibrate under quiescent conditions for 3 days to allow soil settling and initial microbial acclimation. The initial water quality parameters after stabilization were: chemical oxygen demand (COD) 7.7 mg/L, ${\mathrm{NH}}_{4}^{+}$-N 25 mg/L, ${\mathrm{NO}}_{3}^{-}$-N 13.4 mg/L, and TP 3.5 mg/L.

The HFMs used in this study were made of polyvinylidene fluoride (PVDF) with a nominal pore size of 0.2 μm and an inner/outer diameter of 0.6/1.0 mm. Each membrane bundle consisted of 30 individual fibers, with an effective length of 50 cm immersed in the water column, providing a total membrane surface area of approximately 0.047 m^2^ per bundle. Prior to use, the membrane modules were soaked in deionized water for 24 h to remove any residual manufacturing chemicals and then rinsed thoroughly. High-purity H_2_ (>99.999%, *v*/*v*) was supplied from a high-pressure steel cylinder and collected in aluminum foil gas bags (capacity 5 L) fitted with a silicone tubing connector, which served as the H_2_ reservoir for the membrane modules.

### 2.2. Experimental Setup and Operation

**Figure 1:**
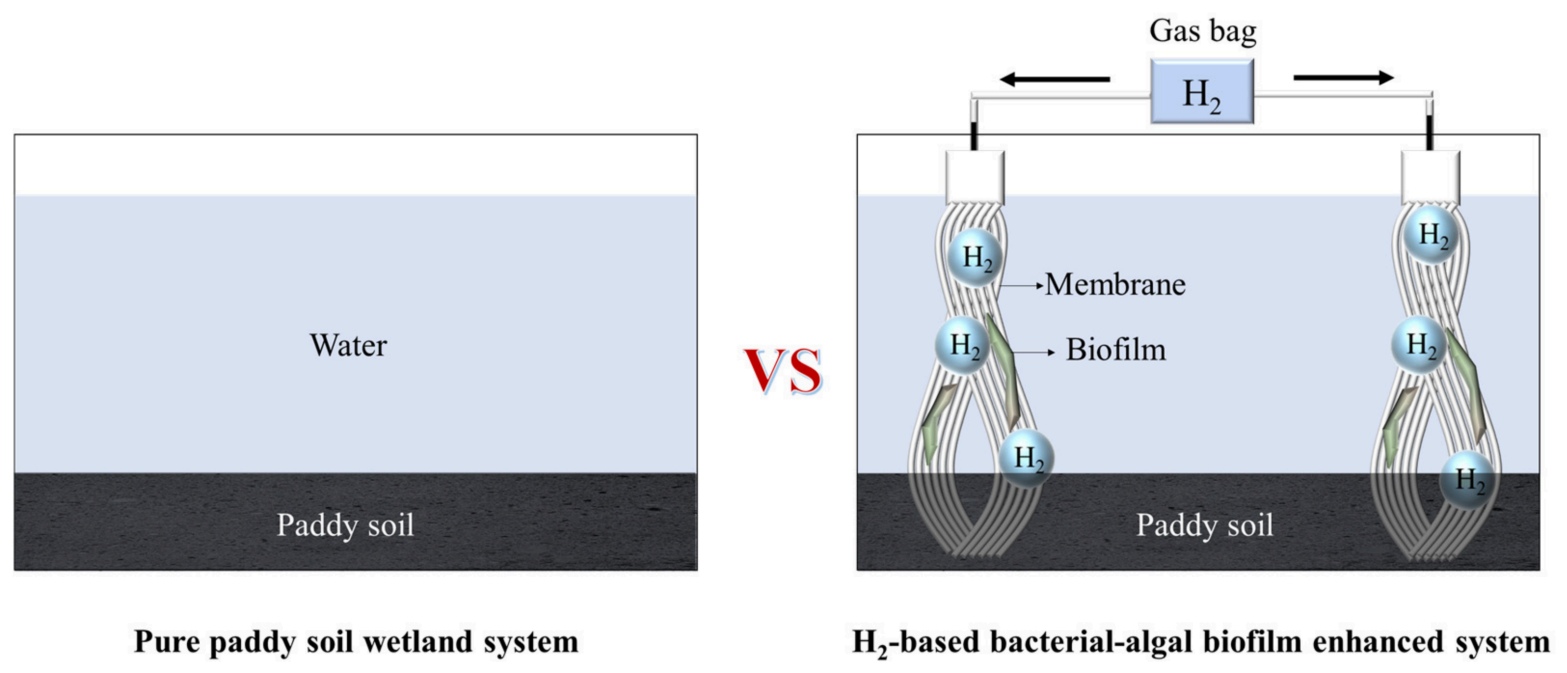
Schematic diagram of the experimental setup.

The experiment was conducted in a sunlight greenhouse located at the Shanghai Academy of Agricultural Sciences from December to February, with natural photoperiod (approximately 10 h light/14 h dark). Two treatment groups, each with three replicates, were established: (1) a pure paddy soil mesocosm without membrane (designated S-1, S-2, S-3), serving as the control; and (2) a H_2_-based bacterial–algal biofilm enhanced system (designated HS-1, HS-2, HS-3), in which the HFM bundle was inserted vertically into the paddy soil with the membrane top connected to the H_2_-filled gas bag (Figure 1). Each gas bag was replaced daily with freshly collected H_2_ to maintain a consistent H_2_ supply pressure (approximately 1.05 atm) throughout the experimental period. After installation, the HFMs were left in place for 7 days under natural illumination to allow the in situ formation of a bacterial–algal symbiotic biofilm on the membrane surfaces; visible greenish biofilms were observed on all HS membranes by day 7.

Following the 7-day biofilm pre-colonization period, the simulated eutrophic water was carefully decanted and replaced with fresh synthetic water of the same composition as described in Section 2.1, marking the start of the formal experiment (day 0). The experiment lasted for 60 days under static batch mode without water exchange or recirculation, except for periodic sampling. No external aeration or mixing was applied, and water loss due to evaporation was compensated weekly with deionized water to maintain a constant water level.

### 2.3. Sampling and Analytical Procedures

Water quality monitoring was conducted at regular intervals throughout the 60-day experiment. On each sampling day (days 0, 3, 7, 10, 14, 21, 28, 35, 42, 50, and 60), water samples were collected from the middle depth (approximately 12 cm below the water surface) of each mesocosm using a 50 mL syringe. The following on-site measurements were performed immediately using a multi-parameter water quality analyzer (Hach HQ 4300, Hach Company, Loveland, CO, USA) equipped with appropriate probes: pH, water temperature (°C), dissolved oxygen (DO, mg/L), and electrical conductivity (EC, μS/cm). Chlorophyll a (Chl-*a*) concentration was determined in situ using a portable chlorophyll fluorometer (Amiscience FluoroQuik, Amiscience Corporation, Fremont, CA, USA).

Grab water samples (approximately 500 mL) were collected in acid-washed polyethylene bottles and transported to the laboratory on ice within 2 h. COD was determined using a COD analyzer (Hach DR3900, Hach Company, Loveland, CO, USA). TP, ${\mathrm{NH}}_{4}^{+}$-N, and ${\mathrm{NO}}_{3}^{-}$-N were measured according to the Standard Methods for the Examination of Water and Wastewater. All nutrient analyses were conducted in triplicate, and results were expressed as mean values.

After each sampling, the water level was restored to the initial depth (23 cm) with deionized water to compensate for both evaporative and sampling losses. The dilution effect was corrected by calculating nutrient concentrations based on the measured water volume at each sampling time.

### 2.4. DNA Extraction and Sequencing

On day 60, immediately after the final water sampling, representative samples were collected for microbial community analysis. From each mesocosm, approximately 2 g of surface paddy soil (0~2 cm depth, designated SN for the S group and HSN for the HS group) was carefully sampled using a sterile spatula and transferred into 2 mL sterile centrifuge tubes. For the HS group, a 2 cm segment of biofilm-attached HFM (designated HSM) was excised from the middle portion of the membrane bundle using sterile scissors and placed into 2 mL sterile tubes. All samples were immediately frozen in liquid nitrogen and subsequently stored at –80 °C until DNA extraction. For each sample type, biological replicates (*n* = 3) were pooled in equal mass prior to DNA extraction to reduce inter-replicate variability and obtain a representative community profile for each treatment.

Total genomic DNA was extracted from 0.5 g of soil or biofilm-scraped membrane samples using the FastDNA^®^ SPIN Kit for Soil (MP Biomedicals, LLC, Solon, OH, USA) according to the manufacturer’s instructions. DNA quality and concentration were assessed by 1% agarose gel electrophoresis and a NanoDrop 2000 spectrophotometer (Thermo Fisher Scientific, Waltham, MA, USA). The extracted DNA was used as template for PCR amplification of the full-length 16S rRNA gene (~1500 bp) using the bacterial universal primers 27F (5′-AGAGTTTGATCMTGGCTCAG-3′) and 1492R (5′-TACGGYTACCTTGTTACGACTT-3′). For eukaryotic community analysis in the biofilm samples, the V4 region of the 18S rRNA gene was amplified using the primers TAReuk454FWD1 (5′-CCAGCASCYGCGGTAATTCC-3′) and TAReukREV3 (5′-ACTTTCGTTCTTGATYRA-3′) [19]. PCR conditions were identical to those described for bacterial 16S amplification, with the annealing temperature adjusted to 56 °C. Amplicons were purified, quantified, and pooled in equimolar concentrations. The 16S amplicons were subjected to SMRTbell^®^ library preparation and sequenced on the PacBio Sequel II platform (Pacific Biosciences of California, Inc., Menlo Park, CA, USA). The 18S V4 amplicons were sequenced on the Illumina platform (Illumina, Inc., San Diego, CA, USA). Sequencing was performed at Beijing Biomarker Technologies Co., Ltd. Subsequent bioinformatics analyses were conducted on the BMK Cloud platform (www.biocloud.net, accessed on 21 September 2026). Detailed sequencing and bioinformatics parameters are provided in Supplementary Text S1. Raw sequence data were submitted to the NCBI SRA under the accession number PRJNA1529377.

### 2.5. Statistical Analysis

Alpha diversity metrics are reported as single composite values because biological replicates were pooled prior to sequencing. No statistical inference was performed on these indices. Differences in physical and chemical indexes between treatments were assessed using one-way analysis of variance (ANOVA), with a significance threshold of *p* < 0.05.

## 3. Results

### 3.1. Water Quality Dynamics and Aqueous Nutrient Concentrations

The experiment was conducted in winter, with temperatures was 12.24 ± 0.01 °C in S system and 12.37 ± 0.15 °C in HS system (Table 1). In addition, the Chl-*a* content in the S system was 72.5 ± 22.5 μg/L, while that in the HS system was 35.8 ± 2.3 μg/L, significantly lower than in the S system.

**Figure 2:**
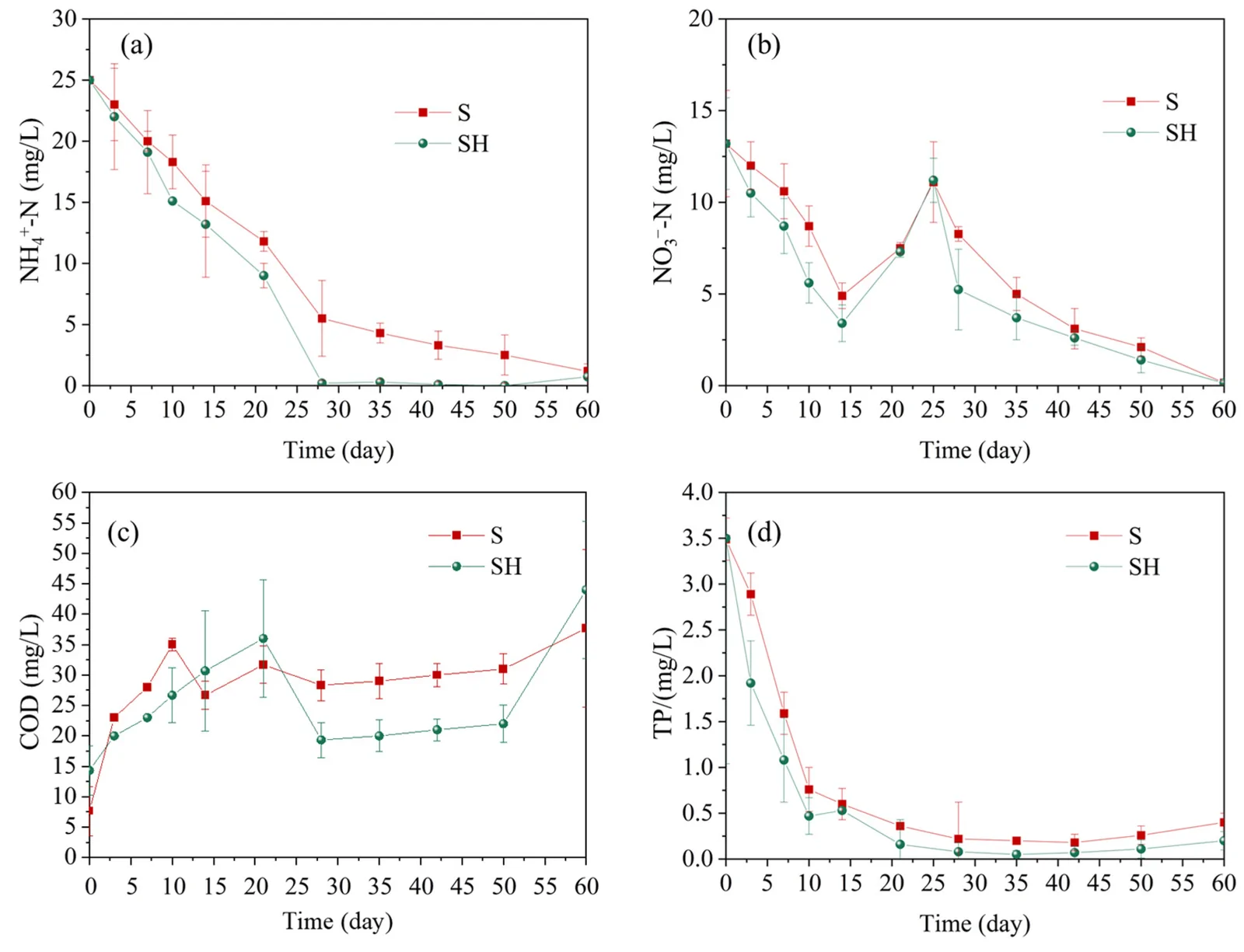
Variations in ${\mathrm{NH}}_{4}^{+}$-N (**a**), ${\mathrm{NO}}_{3}^{-}$-N (**b**), COD (**c**), and TP (**d**) concentrations throughout the operational period.

The nutrient concentrations over time in the HS and S systems are shown in Figure 2. After 30 days of operation under low-temperature conditions, the ${\mathrm{NH}}_{4}^{+}$-N concentration in the HS system decreased from an initial 25.0 mg/L to 0.2 mg/L, and this low level was maintained through day 60, whereas that in the S system remained at 5.5 mg/L. For ${\mathrm{NO}}_{3}^{-}$-N removal, the HS system consistently exhibited lower concentrations than the S system. Considering that COD serves as the electron donor and carbon source for denitrification in paddy soil, and that COD concentrations in both systems ranged from 7.0 to 44.0 mg/L. From day 14 to day 60, the TP concentration in the HS system remained between 0.1 and 0.6 mg/L, significantly lower than the 0.2–0.8 mg/L observed in the S system.

### 3.2. Microbial Community Analysis

#### 3.2.1. Alpha Diversity Analysis

Alpha diversity indices (Ace, Chao1, Simpson 1-D, Shannon, PD_whole_tree, and Coverage) were used to characterize microbial community richness and diversity. The alpha diversity indices for paddy soil microorganisms in different paddy-soil mesocosms are shown in Table 2. The Coverage indices for SN, HSM, and HSN were 0.998, 0.998, and 0.997, respectively, all above 0.99, indicating that the analysis results reliably represent the true community composition.

The Ace and Chao1 indices are used to characterize community richness; higher values indicate greater richness. The Shannon and Simpson 1-D indices reflect community diversity; higher Shannon values and higher Simpson 1-D values indicate higher diversity. The PD_whole_tree index reflects phylogenetic diversity, with higher values indicating greater genetic diversity. The Ace and Chao1 indices for HSN were 931.53 and 935.94, respectively, higher than those for SN, indicating that the H_2_-based bacterial–algal biofilm-enhanced system increased microbial richness. The Shannon and Simpson 1-D indices were similar between SN and HSN. The PD_whole_tree index of HSN was 2.17 higher than that of SN, an increase of 3.44%.

#### 3.2.2. Community Composition of H_2_-Based Bacterial–Algal Biofilm at Species Level

**Figure 3:**
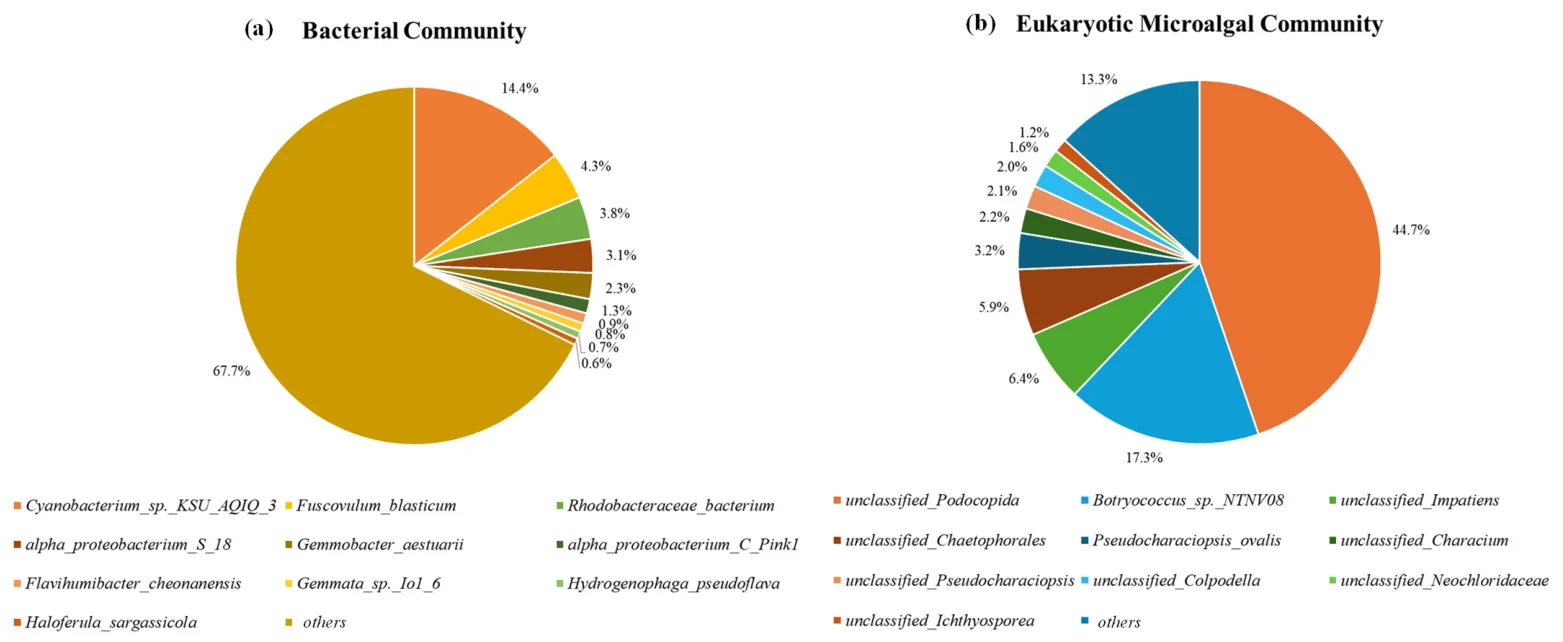
Bacterial (**a**) and eukaryotic (**b**) community composition of H_2_-based biofilm at the species level (TOP10).

As shown in Figure 3a, species-level community analysis revealed that the dominant species in HSM were *Cyanobacterium_sp._KSU_AQIQ_3* (14.4%), *Fuscovulum blasticum* (4.4%), *Rhodobacteraceae_bacterium* (3.8%), *Alpha_proteobacterium_S_18* (3.1%), *Gemmobacter aestuarii* (2.4%), *Alpha_proteobacterium_C_Pink1* (1.3%), *Flavihumibacter cheonanensis* (0.9%), *Gemmata_sp._Io1_6* (0.8%), *Hydrogenophaga pseudoflava* (0.7%), and *Haloferula sargassicola* (0.6%). *Rhodobacteraceae_bacterium* is a typical photosynthetic bacterium involved in photosynthesis and carbohydrate metabolism [20], potentially enhancing the role of planktonic algae in the carbon cycle of water bodies. *Hydrogenophaga pseudoflava* is widely distributed in wastewater treatment plants and exhibits heterotrophic nitrification–aerobic denitrification [21].

To complement the prokaryotic profile obtained from 16S sequencing, the eukaryotic community composition of the H_2_-based biofilm (HSM) was resolved using 18S rRNA gene amplicon sequencing at the species level. As shown in Figure 3b, the eukaryotic community in the biofilm was dominated by *unclassified_Podocopida* (44.7%), a taxon belonging to the class *Podocopida* within the phylum *Arthropoda*, which encompasses benthic ostracods commonly found in aquatic sediments and periphytic biofilms [22]. The second most abundant taxon was *Botryococcus sp._NTNV08* (17.3%), a green colonial microalga (*Chlorophyta*) renowned for its hydrocarbon production and high nutrient uptake capacity [23]. Other notable photosynthetic eukaryotes included *unclassified_Chaetophorales* (5.9%), *Pseudocharaciopsis ovalis* (3.2%), *unclassified_Characium* (2.2%), *unclassified_Pseudocharaciopsis* (2.1%), and *unclassified_Neochloridaceae* (1.6%), all belonging to the *Chlorophyta*. Non-photosynthetic taxa comprised *unclassified_Impatiens* (6.4%), *unclassified_Colpodella* (2.0%), and *unclassified_Ichthyosporea* (1.2%). Collectively, photosynthetic microalgae (primarily *Chlorophyta*) accounted for approximately 34% of the eukaryotic community.

#### 3.2.3. Effect of H_2_-Based Biofilm on the Paddy Soil Microbial Community

**Figure 4:**
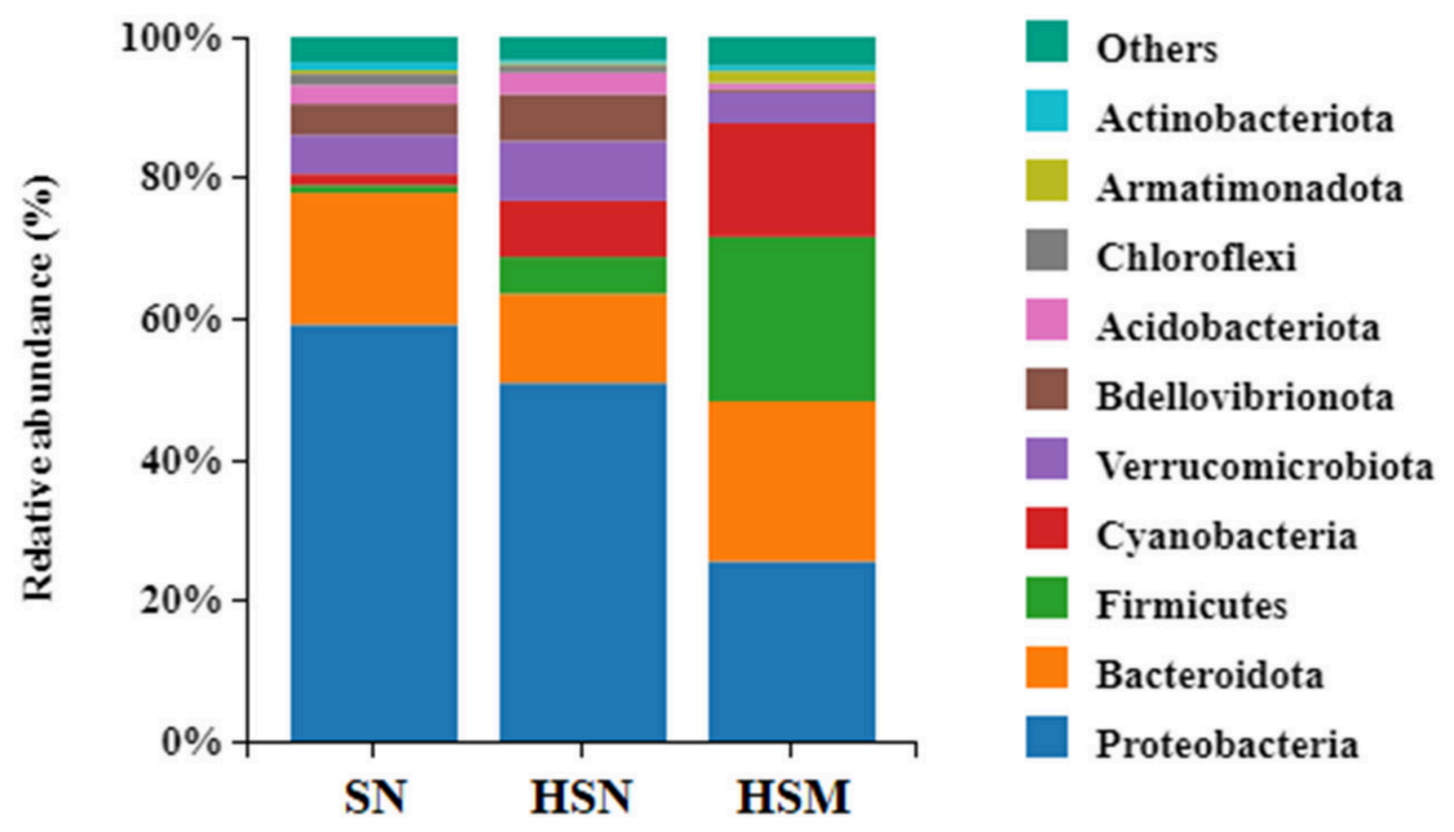
Phylum level microbial community composition of biofilm and paddy soil.

The distribution of microorganisms at the phylum level in biofilm and paddy soil samples is shown in Figure 4. SN represents paddy soil from the S system, HSN represents paddy soil from the HS system, and HSM represents biofilm from the HS system. *Proteobacteria*, *Bacteroidota,* and *Verrucomicrobiota* were the dominant phyla in both paddy soil samples, while *Proteobacteria, Bacteroidota*, *Firmicutes*, and *Cyanobacteria* were dominant in the biofilm. The H_2_-based bacterial–algal biofilm altered the microbial community in paddy soil, with increased abundances of *Firmicutes*, *Cyanobacteria*, *Verrucomicrobiota,* and *Bdellovibrionota* in HSN compared to SN.

**Figure 5:**
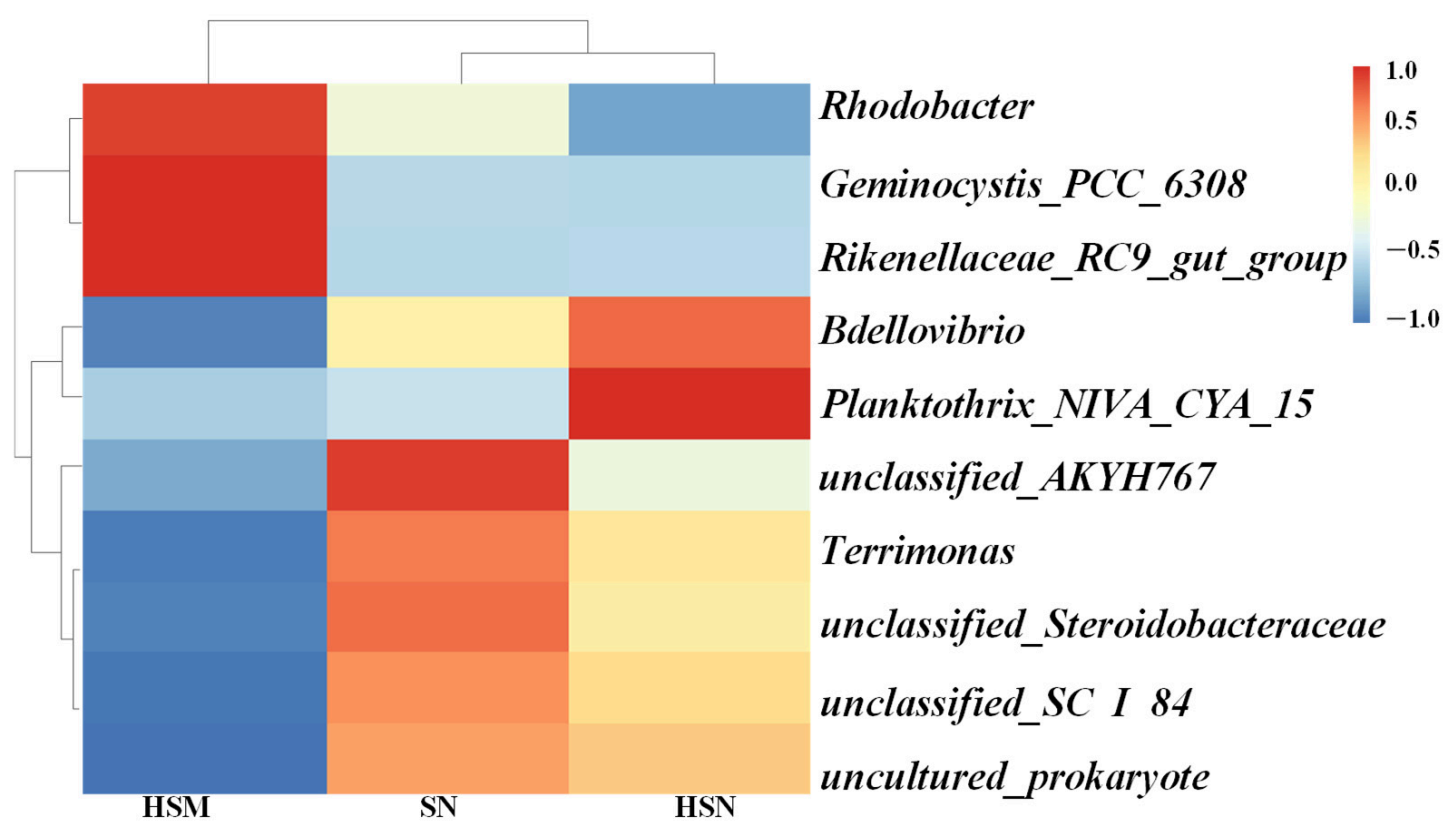
Differences in microbial communities at genus level between biofilm and paddy soil.

Clustering analysis at the genus level (Figure 5) revealed more pronounced differences. HSM was dominated by *Geminocystis_PCC_6308*, *Rikenellaceae_RC9_gut_group*, and *Rhodobacter*. *Geminocystis_PCC_6308* is a functional cyanobacterium for enhancing the degradation of nitrogen and phosphorus [24]. *Rhodobacter* is a common genus in wetland systems with pollutant degradation functions [25]. Influenced by the hydrogen-based bacterial–algal biofilm, the corresponding paddy soil showed *Bdellovibrio* and *Planktothrix_NIVA_CYA_15* as the two most abundant genera. In contrast, the paddy soil from the S system was characterized by *Terrimonas*, *unclassified_AKYH767*, *unclassified_Steroidobacteraceae*, *unclassified_SC_I_84*, and *uncultured_prokaryote*. Among these, *Terrimonas* has been previously reported to have nitrogen and phosphorus removal functions [26], indicating its close association with nitrogen cycling. Although these taxa remained dominant in HSN, their relative abundances decreased, suggesting that a portion of the nitrogen removal and aqueous phosphorus mitigation in the H_2_-based bacterial–algal biofilm paddy-soil mesocosm was performed by the biofilm itself, thereby enhancing nutrient removal under low-temperature conditions.

## 4. Discussion

### 4.1. Nitrogen Removal and Aqueous Phosphorus Reduction

The present study demonstrates that the HS system, in which H_2_ supply, HFMs, and a bacterial–algal biofilm were introduced together, maintained lower aqueous ${\mathrm{NH}}_{4}^{+}$-N, ${\mathrm{NO}}_{3}^{-}$-N, and TP concentrations than the S control under winter conditions (6.2–15.0 °C). The ${\mathrm{NH}}_{4}^{+}$-N concentration in the HS system decreased from 25.0 mg/L to 0.2 mg/L by day 30 and remained at this low level through day 60, whereas the S system still had 5.5 mg/L ${\mathrm{NH}}_{4}^{+}$-N. The HS system also maintained lower ${\mathrm{NO}}_{3}^{-}$-N and TP concentrations than the S system. These results suggest that the combined H_2_–membrane–biofilm system has potential for improving nutrient removal under low-temperature conditions.

The pH was 7.86 ± 0.16 in the S system and 7.88 ± 0.04 in the HS system. Both systems showed a weakly alkaline nature, which may be partly associated with alkalinity generation during denitrification in the paddy soil [27]. pH and DO showed no significant treatment difference, while DO tended to be lower in HS, possibly due to oxygen consumption by the biofilm [12]. The Chl-*a* content in the HS system (35.8 ± 2.3) was significantly lower than that in the S system (72.5 ± 22.5), indicating that the HFMs provided attachment sites for algae, thereby reducing the Chl-*a* content in the water.

The observed decrease in aqueous TP in the HS system is consistent with findings by Yang et al. [28], who reported that algal biofilm-assisted systems enhanced aqueous phosphorus mitigation from domestic wastewater. However, it should be noted that in the absence of biofilm harvesting or a whole-system phosphorus mass balance, a decrease in aqueous TP is not equivalent to removal from the overall system. Phosphorus may have been transferred from the water column to soil or biofilm compartments through adsorption, precipitation, or biological assimilation. Therefore, the TP results should be interpreted as changes in aqueous concentration rather than definitive system-level removal.

### 4.2. Microbial Community Characteristics of the Biofilm and Paddy Soil

The integration of 16S and 18S sequencing data provides a comprehensive view of the photoautotrophic community within the hydrogen-based biofilm, revealing a phylogenetically diverse assemblage of both prokaryotic cyanobacteria and eukaryotic microalgae. While 16S sequencing detected the presence of cyanobacterial genera, 18S sequencing further unveiled the dominance of eukaryotic green algae, particularly *Botryococcus* sp. (17.3%), along with substantial proportions of *Chaetophorales* (5.9%), *Pseudocharaciopsis ovalis* (3.2%), and *Characium-related taxa* (2.2%). This phylogenetic diversity in primary producers carries profound ecological and functional implications for the biofilm system.

The high relative abundance of *Botryococcus* sp. in the biofilm is particularly noteworthy. Members of the genus *Botryococcus* are colonial green algae widely distributed in freshwater and brackish environments, possessing exceptional capabilities for nutrient assimilation, particularly nitrogen and phosphorus uptake [29]. Their colonial morphology, surrounded by a thick extracellular matrix rich in hydrocarbons, provides structural stability to the biofilm and enhances resistance to environmental stressors, including low temperatures [30]. The substantial presence of *Botryococcus* suggests a significant direct pathway for inorganic nutrient sequestration, functioning independently of bacterial metabolic activity, which is particularly advantageous under cold conditions where bacterial enzymatic reactions are kinetically constrained.

The coexistence of eukaryotic green algae (*Chlorophyta*) and prokaryotic cyanobacteria (*Cyanobacterium_sp._KSU_AQIQ_3*) within the same biofilm matrix indicates a dual-source photosynthetic oxygen supply. Cyanobacteria utilize phycobilisomes as light-harvesting antennae, while green algae employ chlorophyll *a* and *b*-containing light-harvesting complexes [31,32]. This biochemical divergence confers distinct light absorption spectra, allowing the two groups to partition the available light spectrum and maximize overall photosynthetic efficiency under varying light intensities and qualities [33]. Furthermore, eukaryotic green algae generally exhibit higher photosynthetic rates at low temperatures compared to cyanobacteria [34], making them particularly valuable in winter paddy-soil mesocosms where temperatures were below 15 °C.

These phylum-level shifts carry important ecological implications. The enrichment of *Firmicutes* in HSN is particularly noteworthy, as members of this phylum are known for their metabolic versatility and resilience to environmental stresses, including nutrient limitation and temperature fluctuations [35]. Many *Firmicutes* species are capable of fermentative metabolism and can utilize complex organic matter, suggesting that the introduction of the biofilm and H_2_ supply may have stimulated anaerobic or facultative anaerobic metabolic pathways in the soil. The increased abundance of *Cyanobacteria* in HSN, albeit at modest levels, indicates that photosynthetic microorganisms may have dispersed from the biofilm to the adjacent soil surface, or that the biofilm-mediated attenuation of water column Chl-*a* (as shown in Table 1) altered light penetration and created favorable conditions for benthic cyanobacterial growth. The elevated *Verrucomicrobiota* abundance is ecologically significant, as this phylum contains many species involved in polysaccharide degradation and carbon cycling, potentially contributing to the mineralization of soil organic matter and release of bioavailable carbon for denitrification [36]. Furthermore, the increase in *Bdellovibrionota* suggests an intensified top-down regulation of bacterial populations, which may accelerate microbial turnover and nutrient remineralization within the soil [37].

Influenced by the H_2_-based bacterial–algal biofilm, the corresponding paddy soil (HSN) showed *Bdellovibrio* and *Planktothrix_NIVA_CYA_15* as the two most abundant genera, a striking shift from the community structure observed in SN. *Bdellovibrio* is a genus of small, motile Gram-negative bacteria that preys on other Gram-negative bacteria by attaching to their cell walls, penetrating the periplasm, and replicating intracellularly, ultimately lysing the host cell [38]. The substantial enrichment of *Bdellovibrio* in HSN suggests that the biofilm may have promoted a trophic cascade in the soil microbial food web. The elevated abundance of potential prey bacteria (e.g., *Proteobacteria* and *Bacteroidota*), stimulated by the continuous H_2_ supply and algal-derived organic carbon, likely provided an abundant food source for *Bdellovibrio* populations. This predatory activity may enhance nutrient regeneration by accelerating microbial biomass turnover, releasing intracellular nutrients (such as ammonium and phosphate) that can be subsequently assimilated by algae or utilized by other bacteria, thereby indirectly contributing to the overall nutrient removal efficiency of the system.

In the S system, nitrogen removal and phosphorus concentration reduction relied almost exclusively on the metabolic activities of soil-resident microorganisms, with *Terrimonas* as the primary driver. In the HS system, however, a substantial portion of the nutrient removal burden was transferred to the H_2_-based bacterial–algal biofilm, which provided additional removal pathways through algal assimilation, hydrogenotrophic denitrification, and enhanced microbial synergy. This functional complementarity relieved the selective pressure on soil indigenous functional taxa, allowing the soil community to diversify and incorporate new trophic interactions that may further enhance ecosystem functioning.

### 4.3. Limitations, Implications and Future Perspectives

Several limitations of this study should be acknowledged. Because H_2_ supply, the HFMs, and the bacterial–algal biofilm were introduced simultaneously in the HS treatment, their individual contributions—the electron-donor effect of H_2_, the physical attachment effect of the membrane, and the biological activity of the biofilm—cannot be separated. In addition, although H_2_ consumption, membrane flux, and dissolved H_2_ concentration were not measured, the enhanced nutrient removal performance of the combined H_2_-based biofilm system relative to the control was clearly demonstrated under the tested conditions. The static batch design was intended to simulate the long hydraulic retention time typical of winter agricultural drainage; however, decreases in aqueous ${\mathrm{NH}}_{4}^{+}$-N, ${\mathrm{NO}}_{3}^{-}$-N, and TP concentrations cannot be equated with permanent removal, as transformation, algal assimilation, soil adsorption, precipitation, and sampling losses may all contribute. Nevertheless, achieving drainage water quality standards remains important for controlling non-point source pollution and the associated eutrophication of receiving waters. Furthermore, this 60-day winter greenhouse batch test did not evaluate long-term stability, year-round operation, H_2_ energy consumption, cost, or scale-up potential. Therefore, the conclusions should be regarded as a proof-of-concept under the specific conditions tested, and future factorial, mass-balance, and pilot-scale studies are needed to resolve these uncertainties.

Despite these limitations, this study provides a proof-of-concept demonstration that the combined H_2_–membrane–biofilm approach has potential for improving winter nutrient removal in paddy soil mesocosms. The integration of hydrogenotrophic denitrification with algal–bacterial symbiosis offers a complementary strategy that may partially compensate for the reduced metabolic activity of indigenous soil microorganisms under low-temperature conditions. The co-occurrence of prokaryotic cyanobacteria and eukaryotic green algae within the biofilm suggests a phylogenetically diverse phototrophic community that may support localized oxygen production and direct nutrient assimilation. To advance this approach toward practical application, future research should focus on: (1) factorial experiments to separate the individual contributions of H_2_, the membrane, and the biofilm; (2) quantification of H_2_ flux and dissolved H_2_ to determine the electron donor dose; (3) full nitrogen and phosphorus mass balances, including gaseous N products and soil/biofilm nutrient pools; (4) functional validation using metagenomics, metatranscriptomics, and process-rate measurements; and (5) long-term, continuous-flow, pilot-scale testing across multiple seasons to evaluate stability, energy consumption, and cost-effectiveness.

## 5. Conclusions

(1) The HS system achieved lower aqueous ${\mathrm{NH}}_{4}^{+}$-N, ${\mathrm{NO}}_{3}^{-}$-N, and TP concentrations than the S system under low temperature conditions. After 60 days of operation, the HS system reduced ${\mathrm{NH}}_{4}^{+}$-N from 25.0 mg/L to 0.2 mg/L, while the S system only reached 5.5 mg/L. Additionally, the HFMs provided attachment sites for algae, shifting algal biomass from planktonic to attached growth and reducing water column Chl-*a* from 72.5 ± 22.5 to 35.8 ± 2.3 μg/L.

(2) The lower aqueous nutrient concentrations in the HS system are consistent with a coupled nitrification–denitrification process. 16S sequencing identified cyanobacterium *Geminocystis_PCC_6308*, along with *Rikenellaceae_RC9_gut_group* and *Rhodobacter*, as the dominant bacterial components in the biofilm, while 18S sequencing revealed that eukaryotic green algae—particularly *Botryococcus* sp. (17.3%), *Chaetophorales* (5.9%), and *Pseudocharaciopsis ovalis* (3.2%). Concurrently, the soil microbial community shifted from dominance of indigenous functional taxa (e.g., *Terrimonas* and *unclassified_AKYH767*) toward enrichment of *Firmicutes*, *Verrucomicrobiota*, and predatory *Bdellovibrionota*, indicating a restructuring of the soil micro-food web in response to biofilm colonization.

(3) This study demonstrates that the H_2_-based bacterial–algal biofilm system offers a promising technological strategy for enhancing winter performance of paddy-soil mesocosms. By integrating hydrogenotrophic denitrification with algal–bacterial symbiosis within a single system, this approach may help address the challenge of low-temperature inhibition on nitrogen removal and phosphorus concentration reduction, contributing to the broader understanding of soil–microbe–biofilm interactions in engineered ecosystems.

## Figures and Tables

**Table 1:** Physical and chemical indexes of water body.

	pH	DO (mg/L)	Electrical Conductivity (µS/cm)	Water Temperature (°C)	Chl-*a* (μg/L)
S	7.86 ± 0.16	9.61 ± 0.86	1160 ± 16	12.24 ± 0.01	72.5 ± 22.5
HS	7.88 ± 0.04	8.91 ± 0.32	1207 ± 53	12.37 ± 0.15	35.8 ± 2.3 *

Note: Values are time-averaged means ± SD of three independent mesocosms per treatment (*n* = 3). Water temperature ranges were 6.2–15.0 °C (S) and 6.8–15.0 °C (HS). *, *p* < 0.05 (one-way ANOVA).

**Table 2:** Alpha diversity index of microbial communities in different samples.

Group	Sample	ACE	Chao1	Simpson 1-D	Shannon	PD_Whole_Tree	Coverage
S	Paddy soil (SN)	851.28	883.81	0.96	6.68	63.11	0.998
HS	Biofilm (HSM)	907.10	949.00	0.97	7.51	61.33	0.998
	Paddy soil (HSN)	931.53	935.94	0.97	6.89	65.28	0.997

## Data Availability

The raw data supporting the conclusions of this article will be made available by the authors on request.

## Supplementary Materials

The following supporting information can be downloaded at: https://www.mdpi.com/article/10.3390/microorganisms14102230/s1, Text S1: Detailed sequencing and bioinformatics analysis.
