# Supplementary material for: A Hydrogen-Based Bacterial–Algal Biofilm for Winter Nutrient Mitigation in a Paddy Soil Mesocosm: A Proof-of-Concept Study
Source: Microorganisms. 2026 Oct 3;14(10):2230. doi: 10.3390/microorganisms14102230 (PMC13707709; doi:10.3390/microorganisms14102230)
Supplement: Supplementary file 1 [file microorganisms-14-02230-s001.zip › microorganisms-4584689-supplementary.pdf]

# Supplementary Materials

**A Hydrogen-Based Bacterial-Algal Biofilm for Winter Nutrient Mitigation in a Paddy Soil Mesocosm: A Proof-of-Concept Study**

Si Pang [1,3], Naxin Cui [1,3], Min Cai [1,3], Li Zhou [1,3], Yu Tian [2,*] and Xu Zhang [1,2,3,*]

[1] Eco-Environmental Protection Research Institute, Shanghai Academy of Agricultural Sciences, Shanghai 201403, China

[2] State Key Laboratory of Urban-Rural Water Resources and Environment, Harbin Institute of Technology, Harbin 150090, China

[3] Shanghai Engineering Research Centre of Low-Carbon Agriculture, Shanghai 201403, China

* Correspondence: zhangxu@saas.sh.cn; hittianyu@163.com

**Summary of SI：**

- Pages: 3

**Supplementary Text S1.** Detailed sequencing and bioinformatics analysis

### S1.1. Amplicon sequencing

PCR reactions were performed in a 25 μL volume containing 1× PCR buffer, 1.5 mM $MgCl_2$, 0.2 mM each dNTP, 0.4 μM each primer, 1.0 U of high-fidelity DNA polymerase, and 10 ng of template DNA. The thermal cycling protocol for 16S was: initial denaturation at 94°C for 3 min; 30 cycles of 94°C for 30 s, 55°C for 30 s, and 72°C for 90 s; and a final extension at 72°C for 10 min. For 18S, the annealing temperature was adjusted to 56°C. Amplicons were purified using Agencourt AMPure XP beads (Beckman Coulter, USA), quantified with a Qubit 3.0 fluorometer (Invitrogen, USA), and pooled in equimolar concentrations. For 16S, SMRTbell libraries were prepared and sequenced on the PacBio Sequel II platform (Pacific Biosciences, USA). For 18S, libraries were prepared and sequenced on the Illumina platform (Illumina, USA). All sequencing was performed at Beijing Biomarker Technologies Co., Ltd.

### S1.2. Raw data processing and read generation

For 16S, raw subreads were processed using SMRT Link v8.0 (Pacific Biosciences). Circular consensus sequencing (CCS) reads were generated with minPasses ≥5 and minPredictedAccuracy ≥0.9. CCS reads were demultiplexed using lima v1.7.0 with default parameters. Primers were removed using cutadapt v2.7 with a maximum mismatch rate of 20%. CCS reads outside 1,200–1,650 bp were discarded. Chimeric sequences were removed using UCHIME v8.1.

For 18S, raw paired-end reads were quality-filtered using Trimmomatic v0.33 with a 50-bp sliding window and an average quality threshold of 20, truncating reads from the window start to the end when the average quality fell below 20. Primer sequences were identified and removed using Cutadapt v1.9.1 with a maximum mismatch rate of 20% and minimum coverage of 80%. Paired-end reads were merged using USEARCH v10.0 with a minimum overlap of 10 bp, minimum overlap similarity of 90%, and a maximum of 5 mismatches. Reads outside the expected 18S amplicon length range (300–500 bp) were discarded. Chimeric sequences were removed using UCHIME v8.1.

### S1.3. OUT construction and taxonomic assignment

For both 16S and 18S, high-quality reads were clustered into operational taxonomic units (OTUs) at 97% similarity using USEARCH v10.0. OTUs with a total abundance <0.005% of all sequences were discarded. Representative OTU sequences were taxonomically assigned using QIIME2 v2020.6. For 16S, assignment was performed against the SILVA Release 138 database using classify-consensus-blast (minimum similarity 90%, minimum coverage 90%, minimum consensus 51%) and/or classify-sklearn (confidence 0.7). For 18S, assignment was performed against the PR2 v5.0.0 database with a confidence threshold of 0.7, as used in the

company report. If the company confirms that PR2 was not used, replace PR2 with the actual database listed in the final report, e.g., SILVA Release 138 or UNITE Release 8.0.

**S1.4. Low-abundance filtering and normalization**

OTUs with a total abundance <0.005% of all sequences were removed prior to downstream analysis. For alpha and beta diversity analyses, samples were rarefied to the minimum sequencing depth across all samples (16S: 40750 reads; 18S: 61500 reads). Alpha diversity metrics (Ace, Chao1, Shannon, Simpson, PD_whole_tree, and Good's coverage) were calculated on the rarefied dataset. For 16S, beta diversity was assessed using weighted and unweighted UniFrac, Bray-Curtis, and binary Jaccard distances. For 18S, beta diversity was assessed using Bray-Curtis and binary Jaccard distances. Beta diversity patterns were visualized by PCoA, NMDS, and UPGMA.
